# Complementary prognostic values of stress myocardial perfusion and late gadolinium enhancement imaging by cardiac magnetic resonance in patients with suspected myocardial ischemia

**DOI:** 10.1186/1532-429X-11-S1-O18

**Published:** 2009-01-28

**Authors:** Kevin Steel, Ryan Broderick, Vijay Gandla, Eric Larose, Frederick Resnic, Michael Jerosch-Herold, Kenneth Brown, Raymond Y Kwong

**Affiliations:** 1 Wilford Hall Medical Center, San Antonio, TX USA; 2 Brigham and Women's Hospital, Boston, MA USA; 3 Quebec Heart Institute, Quebec City, QC Canada; 4 University of Vermont College of Medicine, Burlington, VT USA

**Keywords:** Myocardial Perfusion, Cardiac Magnetic Resonance, Myocardial Perfusion Imaging, Late Gadolinium Enhancement, Dipyridamole

## Background

Recent studies have demonstrated the prognostic implication of CMR myocardial perfusion imaging (CMRMPI) in a clinical setting. Apart from detecting reversible perfusion defect from flow-limiting coronary stenosis, CMR late enhancement imaging (LGE) is currently the most sensitive method in detecting clinically unrecognized subendocardial infarction (UMI) from prior ischemic injury. We therefore tested the hypothesis that, characterization of these 2 processes from coronary artery disease (CAD) by CMR can provide complementary patient prognostic values.

## Methods and Results

**Figure 1:**
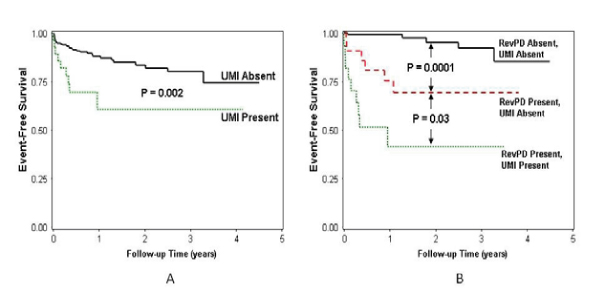
**Kaplan-Meier curves illustrating**. A) the prognostic implications of UMI alone and B) the complementary prediction of MACE by RevPD and UMI, in patients without any history of MI.

**Figure 2:**
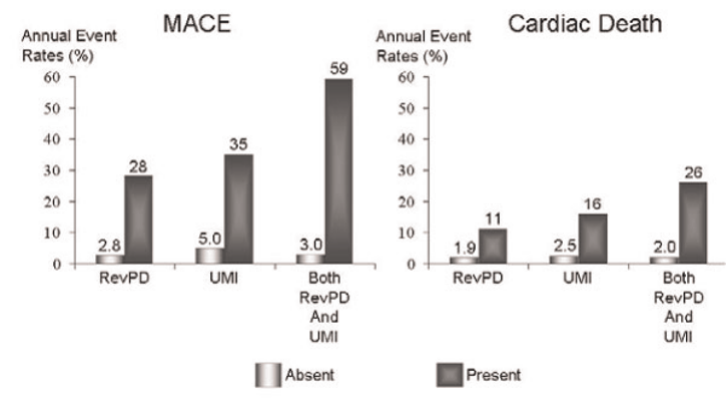
**Annual event rates of MACE and cardiac death by RevPD, UMI, and both RevPD and UMI**.

We performed CMR on 254 patients referred with symptoms suspicious of myocardial ischemia. Rest and vasodilator (adenosine or dipyridamole) stress first-pass CMRMPI images were obtained and followed by LGE imaging. All CMRMPI images were interpreted for reversible perfusion defects (RevPD) using the 16-segment nomenclature and graded segmental LGE in a separate session. The readers were blinded to any clinical outcome in either session. At a median follow up of 15 months, 13 cardiac deaths and 26 nonfatal events occurred. RevPD was the strongest multivariable predictor to MACE, demonstrating a > 8-fold hazard increase to MACE (P < 0.0001) and a > 4-fold increase to cardiac death (P = 0.02). Adjusted to the effects of RevPD, LGE maintained a > 2-fold adjusted hazards with MACE (adjusted HR 2.38, P = 0.03). In 198 patients without any history of MI, presence of RevPD and UMI by LGE provided complementary prognostic information after adjusting to each other's effects. Figures 1 and 2.

## Conclusion

Reversible myocardial perfusion and evidence of MI, assessed by CMRMPI and LGE, respectively, provide incremental long-term patient prognostic information.

